# Childhood Obesity — What We Can Learn From Existing Data on Societal Trends, Part 2

**Published:** 2005-03-15

**Authors:** Roland Sturm

**Affiliations:** RAND

## Abstract

The number of overweight and obese youth has increased in recent decades, yet few data assess how the lives of children have changed during the "obesity epidemic." Part 1 of this two-part study discussed trends in time use, studying at home, and media use. Part 2 focuses on transportation, physical education, and diet.

Walking or biking for transportation can expend a large amount of energy, but active transportation is not a major source of physical activity for youth, averaging eight minutes a day in 2001, with little change over the past few decades. For adolescents, there was no clear trend in physical education during the past decade, but there are no data for after-school and day-care programs, which have become more important as children spend more time away from home. For younger children, time spent in organized sports and outdoor activities increased by 73 minutes per week between 1981 and 1997.

Eating as a primary activity declined, suggesting a shift toward snacking or eating as a secondary activity. Statistically significant trends exist for carbohydrate intake, especially for chips/crackers/popcorn/pretzels (intake tripled from the mid-1970s to the mid-1990s) and soft drinks (intake doubled during the same period). Price and income data suggest possible economic reasons for these changes. The percentage of disposable income spent on food has declined continuously, and almost all of the decline has been represented by food consumed at home, yet today's disposable income buys more calories than it has in the past. Relative prices have encouraged shifts across food types. From a baseline of 100 during 1982–84, the price index for fresh fruit and vegetables increased to 258 by 2002 (far exceeding general inflation), whereas the price index for soft drinks increased only to 126 by 2002 (below general inflation).

## Introduction

The number of overweight and obese youth has been increasing, reflecting changes in social and environmental factors that need to be understood and modified for effective prevention (1-3). This two-part report reviews some data available to track how the lives of children have changed during the "obesity epidemic." Part 1 reviewed changes in time use, homework, and media use; Part 2 reviews transportation, physical education (PE), and diet.

Active transportation, such as walking or biking, can expend a large amount of energy, and it has been hypothesized that increased suburbanization reduces walking and biking. Only recently have transportation patterns and urban design in relation to physical activity and health attracted interest. Although research has been limited to cross-sectional comparisons and adults, it has shown an association between increases in sprawl and decreases in leisure time and utilitarian walking and increases in body mass index and chronic health conditions (4,5). Only the National Household Travel Survey (NHTS) provides national data for youth travel; it is discussed in the next section of this paper.

We saw in Part 1 that children now spend more time away from home than in the past. As a result, physical activity in school, after-school programs, and day-care settings plays a more important role in determining physical activity levels of children. Limited data are available on physical activity among high school students, which are discussed below, and there are few other data.

Many of the most prominent hypotheses on weight gain address changes in food and diet and the roles of such factors as soft drinks, vending machines, snacks, fast food, and portion sizes. In contrast to the availability of data on PE and transportation, there is a vast scholarly literature on dietary change, and this paper cannot do justice to such a broad area. An Institute of Medicine report on preventing childhood obesity provides a more comprehensive list of citations (6). In addition, the U.S. Department of Agriculture's (USDA's) Economic Research Service regularly updates macroeconomic data in the Food Consumption Data System (7). Nevertheless, interesting data are available on relative food price changes and trends in the eating patterns of children that, while well known among researchers in the nutrition field, do not appear to be widely known among the broader research community interested in child health.

## Transportation

Transportation is an important part of everyday life. American adults spend more than 10 hours per week traveling, about equally divided among transportation related to occupation (work commute), home activities (child care/shopping/personal care), and leisure-time activities (8). Adult transportation and leisure time have increased at the expense of occupation and household activities, with particularly large increases before 1985.

The only national data that provide somewhat comparable data over multiple years are the Nationwide Personal Transportation Surveys (NPTS), now called the NHTS, conducted in 1969, 1977, 1983, 1990, 1995, and 2001 by the U.S. Department of Transportation (9). Walking is generally more underestimated than other transportation modes, and only the 2001 survey probed for walking trips. For our purposes, we use only three years: 1977, 1990, and 2001. There is no walking information in the 1969 survey; the 1983 sample was small and not representative for children; and the 1995 data neglected walking and biking trips. Although the data are intended to be nationally representative, the consistency of methods and quality of data collection is noticeably lower than for the time-use and education data reported in Part 1 of this paper. Thus, while the transportation data discussed in this article are likely to indicate true secular changes, readers should view them with greater skepticism. We do not show statistical significance tests because the main source of uncertainty stems from design changes, not from statistical uncertainty, and statistical tests would suggest more precision than there really is.

The Table shows the share of trips (one-way short- or long-distance travel) by transportation mode for children aged five to 15 years. No clear trend is visible, especially no precipitous decline of walking. Somewhat surprising is the very large increase in the role of school buses between 1977 and 1990, which subsequently declined (and could very well be a methods artifact).

**Figure 1:**
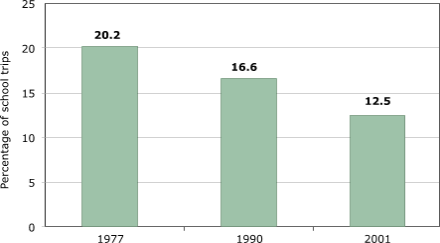
Walking to school as percentage of school trips among U.S. children aged five to 15 years. Author's analysis based on data from National Personal Transportation Survey for 1977 and 1990 and the National Household Travel Survey for 2001 (9).

Travel to and from school is a regular, predictable, and important part of children's travel, even if these trips account only for a minority of all trips. Policy influences how children travel to school more than it influences other travel modes because of decisions on school location or busing, so policy offers better opportunities for interventions in school transportation than it does in other areas. Figure 1 shows a clear and significant decline in the percentage of walking trips for children aged five to 15 years. The trend is slightly stronger for adolescents, dropping from 20.9% in 1977 to 13.6% in 1990 to 10.9% in 2001 (data on adolescents only not shown). Because the total number of school trips does not change much, it seems safe to say that physical activity associated with getting to and from school has declined, despite data limitations. These are probably the best available numbers for youth transportation choices, but they do not provide a complete picture and in isolation are even misleading because school trips are only a small part of total trips, and other trips have not remained constant.

**Table:** 

**Figure 2:**
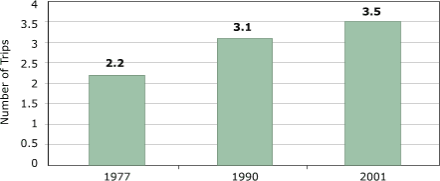
Total number of daily trips among U.S. children aged five to 15 years. Author’s analysis based on data from National Personal Transportation Survey for 1977 and 1990 and the National Household Travel Survey for 2001 (9).

The overall role of walking and biking is more difficult to assess because physical activity depends on the time and distance spent walking or biking. One reliable fact is that the total number of daily trips made by children has substantially increased (Figure 2), so even a decline in the share of walking or biking does not automatically translate into a decline in physical activity. The increase in the number of trips is not surprising because the time-use section in Part 1 showed that children are now spending more time away from home than in the past.

**Table:** 

**Figure 3:**
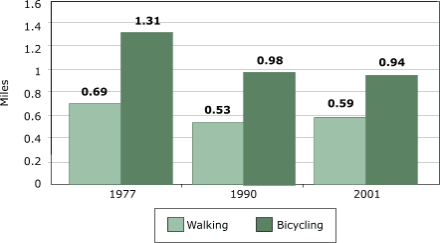
Average trip length (in miles) among U.S. children aged five to 15 years. Author’s analysis based on data from National Personal Transportation Survey for 1977 and 1990 and the National Household Travel Survey for 2001 (9).

Trip distances are not necessarily fixed, and increased suburban sprawl could increase the distance of walking or biking trips (because all destinations are now farther away) or decrease it (if the substitution of driving for walking or biking overcompensates). Here the data quality becomes more questionable. In the NPTS/NHTS, we see little evidence of changes in walking-trip length for children, but biking distances declined noticeably, from about 1.3 miles in 1977 to 0.9 miles in 2001 (Figure 3).

**Table:** 

**Figure 4:**
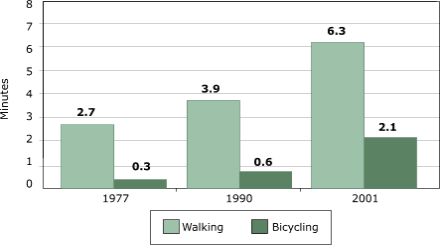
Average active travel time (in minutes) among U.S. children aged five to 15 years. Author's analysis based on data from National Personal Transportation Survey for 1977 and 1990 and the National Household Travel Survey for 2001 (9).

The best metric for physical activity is total active travel time, which incorporates changes in number of trips, distances, and travel mode (Figure 4). Based on the available data, active travel time appears to have increased, a consequence of the increase in the total number of trips, even as walking to school unambiguously declined.

**Table:** 

The magnitude of active travel time is important, however. The highest estimates for active travel time (which were recorded for 2001) add up to only about eight minutes of walking and biking combined per day. So even with a 50% increase, the energy expenditure associated with active travel time would be no more than the energy equivalent of a half-can of soft drink.

On the positive side, interventions that increase walking to school could effect a large relative change. If an additional one quarter of children were to walk to school — not an entirely unrealistic scenario because enough children live within walking distance of their schools — total active travel time could increase by 50% nationwide.

Transportation offers promising interventions for other reasons. Transportation patterns depend on public goods, and large externalities are associated with individual automobile use (10). The growth of traffic and accompanying changes in land use reduce incentives to walk or bike because nearby destinations are disappearing and because of perceived (and actual) danger. The absence of good time-series data on the travel patterns and mobility needs of children and the focus on planning for automobiles by federal, state, and local transit and planning agencies indicate neglect. New policies and investments that make cities safer and more convenient for walking and biking may be economically efficient aside from the benefit to the health and mobility of children (10).

## Physical Education

Children spend many hours in school, making PE programs in schools a potentially important channel through which physical activity and fitness may be promoted among young children (11,12). Arguably as important, though rarely studied or discussed, is physical activity in day care and after-school programs, where children are spending far more time now than two decades ago.

In 1998, 16% of kindergartners received daily PE instruction in school, and approximately 13% received PE instruction less than once a week or never (13). Results of the 2001 Youth Risk Behavior Surveillance System (YRBSS), a national school-based survey of ninth- to 12^th^-graders conducted by the Centers for Disease Control and Prevention (CDC) show that nearly one half (45%) do not play team sports during the year; nearly one half (48%) are not enrolled in a PE class; and PE enrollment drops from 74% for ninth-graders to 31% for 12^th^-graders (14).

Trend data on changes are more difficult to find. The YRBSS goes back only as far as 1991 and then only for a few variables. The quality of these nationally representative data is excellent, but limitations stem from the short time period, the small number of PE items available in all years, and a narrow target population that excludes younger children. The consistency of the YRBSS data, however, is much better than the consistency of the transportation data.

**Figure 5:**
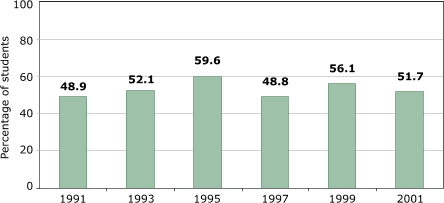
Percentage of U.S. high school students who attended physical education class one or more days during an average school week. Data from the Youth Risk Behavioral Surveillance System, Centers for Disease Control and Prevention (14).

Figure 5 shows the percentage of high school students who attended PE classes at least once a week. Little evidence suggests a continuous trend in either direction. If we were to fit a linear trend to these averages, it would suggest an increase in participation by about two percentage points per decade — not exactly a major increasing trend, but certainly not evidence of a decline either. The absolute level, however, could be considered disappointingly low: almost half of U.S. high school students do *not *receive regular PE in school.

**Table:** 

**Figure 6:**
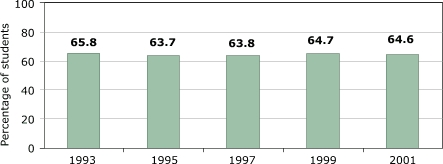
Percentage of U.S. high school students who exercised or participated in physical activities that made them sweat and breathe hard for at least 20 minutes on three or more of past seven days. Data from the Youth Risk Behavioral Surveillance System, Centers for Disease Control and Prevention (14).

Despite fluctuations in reported PE participation, the percentage of high school students who get enough physical activity to satisfy minimum guideline levels is essentially constant (the difference between the highest and lowest annual numbers is only two percentage points) and much higher (Figure 6) than the rates of high school students receiving regular PE in school. This variable includes physical activity outside school. Even if total participation is less than optimal, there is no evidence for declining exercise levels.

**Table:** A text description of this chart is also available.

	**Percentage of students**
**1993**	65.8
**1995**	63.7
**1997**	63.8
**1999**	64.7
**2001**	64.6

**Figure 7:**
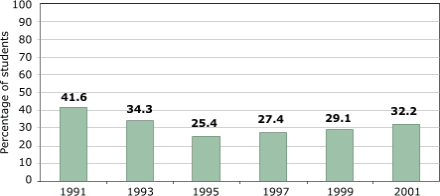
Percentage of U.S. high school students attending daily physical education classes. Data from the Youth Risk Behavioral Surveillance System, Centers for Disease Control and Prevention (14).

Only one variable is inconsistent with the physical activity data described above: namely, the percentage of students participating in daily PE (Figure 7). Participation rates were highest in 1991 and then dropped quickly to bottom out in 1995, followed by a significant and continuous increase since then. The 1991 number itself seems to be out of line with other years and could potentially be an issue of methodology — national changes of that magnitude rarely happen so quickly nor do they immediately reverse themselves. Numbers for 1993 and 2001 are similar.

**Table:** 

How effective are school PE programs in preventing obesity and promoting physical activity? School boards are receiving mixed messages about PE. On one hand, government organizations like the CDC recommend that all schools require daily PE for all students from kindergarten through 12^th^ grade. On the other hand, the predominant conclusion emerging from research studies is that typical school PE is of low quality when compared with ideal PE instruction. School boards, principals, and teachers facing other competing goals, especially academic achievement, may conclude that if existing PE is of limited value, it should be abolished or at least reduced in favor of other academic instruction. However, PE in elementary schools as currently implemented nationwide (and not ideal instruction) plays an important role in containing excess weight gain among girls (13).

## Diet

We can examine trends in dietary change for children by using data from the Continuing Survey of Food Intakes by Individuals (CSFII) 1989–91, 1994–96, and 1998 and the Nationwide Food Consumption Survey 1977–78. Enns et al have published the results of these surveys for children aged six to 11 years (15). There are only two strong and consistent trends. One, the intake of chips/crackers/popcorn/pretzels roughly tripled from the mid-1970s to the mid-1990s: from five grams (1977–78) to nine grams (1989–91) to 14 grams (1994–96, 1998) per day for girls and from five grams to nine grams to 15 grams for boys. Two, the intake of soft drinks roughly doubled during the same period: from 105 grams to 136 grams to 200 grams per day for girls and from 112 grams to 169 grams to 217 grams per day for boys. Other researchers found parallel changes for all age groups, and trends appear similar for different age groups (16). While increased snacking is likely a main cause for the shift across foods, there also has been a shift to larger portion sizes (17,18).

Some researchers believe that high-fructose corn syrup or added caloric sweeteners play an important role in the development of obesity worldwide (19,20). For a typical soft drink, 100 mL (or less than one third of a 12-oz can) has 10.7 g of sugar and provides 43 kcal of energy. This energy value corresponds to the energy expenditure of about eight minutes of walking for an adult and to the daily average that is reported as active travel time among children aged five to 15 years in the 2001 NHTS. Thus, the increase in soft drink consumption alone appears to be at least equal to the total energy expenditure associated with children's active travel in 2001. That this trend in soft drink consumption could be a factor in weight gain is also consistent with cross-sectional data that show an association between the consumption of sugar-sweetened drinks and obesity after controlling for observable characteristics (21). Soft drink consumption is also negatively related to milk, fruit, and vegetable consumption and positively related to higher calorie intake (22-24).

**Figure 8:**
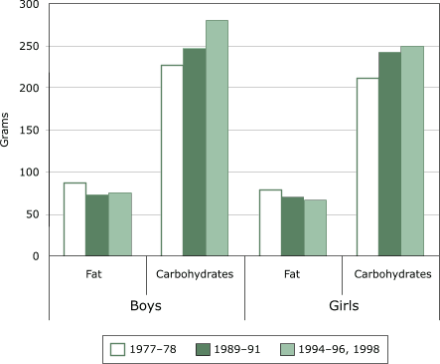
Daily fat and carbohydrate intake in grams per day for U.S. boys and girls aged six to 11 years. Data from Continuing Survey of Food Intakes by Individuals for 1989–91, 1994–96, and 1998 and Nationwide Food Consumption Survey 1977–78, published by Enns et al (15).

Two significant trends are apparent in the share of energy from fat and carbohydrates. The share of energy from fat fell for both boys and girls, and the share of energy from carbohydrates increased (15). Figure 8 shows changes in grams. Fat intake decreased by about 100 kcal or less, but carbohydrate intake increased by about 150 to 200 kcal. The point estimate of total energy intake in the 1990s was higher than in 1977–78, but the data cannot reject the hypothesis of no change.

**Table:** 

**Figure 9:**
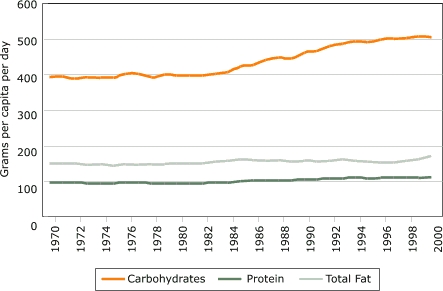
U.S. food supply of macronutrients in grams per capita per day, 1970–2000. Data from Food Consumption Data System, Economic Research Service, U.S. Department of Agriculture (7).

An alternative data series is provided by the USDA's Food Consumption Data System, but it cannot identify trends for subgroups because it is composed of macroeconomic data (Figure 9). Calories per capita remained relatively constant from 1970 until the mid-1980s but then increased. Consistent with the CSFII data, the energy increase is derived almost exclusively from carbohydrates.

Price and income data may be important because they shed light on underlying economic trends. The percentage of disposable income spent by Americans on food has continuously declined since the end of World War II, even as it bought more calories. Almost all of the decline is derived from food prepared and consumed at home; the share of disposable income for food away from home stayed relatively constant. In 1970, Americans spent one third of their food dollars on food away from home; this amount grew to 39% in 1980, 45% in 1990, and 47% in 2001. Away-from-home foods tend to be more energy-dense and contain more fats and sugars than foods at home. USDA researchers have calculated that if food away from home had the same average nutritional densities as food at home in 1995, Americans would have consumed 197 fewer calories per day and reduced their fat intake to 31.5% of calories (instead of the actual 33.6%) (25).

With increasing income, people are shifting to more convenient food away from home. Demographic reasons explain this shift as well. Increased numbers of smaller households (resulting from lower fertility rates) and increased numbers of single-parent households enjoy fewer economies of scale in home-food production than larger families. Preparation of an in-home meal involves a fixed time that differs little with the number of persons served, whereas eating out involves the same marginal costs for each person. This difference in "technology" combined with demographic changes alone would have shifted incentives toward fewer meals prepared at home. In addition, relative price changes have made the consumption of prepared foods cheaper compared with the time costs of preparing food at home and cleaning up.

What is not clear is why the location of consumption should so dramatically alter nutritional content. Lack of information at the point of consumption is probably part of the reason, although this argument would only apply to adults who are presumed to be able to make rational decisions. If adults lack information about nutritional content at the point of consumption, it is not surprising that competition takes place among factors that consumers can evaluate easily (at least with repeat purchases): price, amount, and taste. This type of market failure is well known to economists since Nobel Laureate George Akerlof's "lemon paper" (26). Akerlof argued that if quality is an important dimension but cannot be assessed by a buyer, competition will take place on price and other observed characteristics (e.g., portion size) and drive out higher-quality products even if they would be preferred by buyers with more complete information. When informational problems are sufficiently severe, regulation is needed for an efficient market. Requiring easily available and understandable information about the nutritional content of prepared meals at the point of consumption might address this informational problem.

**Figure 10:**
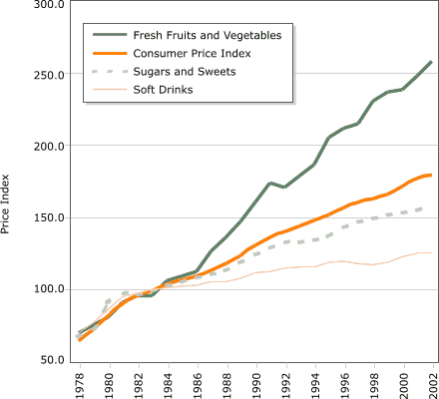
Relative price changes for fresh fruits and vegetables, sugars and sweets, and soft drinks, using the period 1982–84 as the baseline (index = 100), 1978–2002. Data from Food Consumption Data System, Economic Research Service, U.S. Department of Agriculture (7).

Another economic trend shown by data collected by the USDA's Economic Research Service (7) also shifts incentives in a direction that does not promote healthier eating patterns. Figure 10 shows relative price changes, using the period 1982–1984 as the baseline (index = 100) for each series. While the consumer price index increased to 180 by 2002, the price index for fresh fruit and vegetables increased to 258. In contrast, sugars, sweets, fats and oils became relatively cheaper than other goods, and their prices increased less than the consumer price index (data not shown for fats and oils). With a 2002 price index of 126, soft drinks were among the items that became (relatively) the cheapest.

**Table:** 

Detailed data exist on lunches and breakfasts offered in schools participating in the National School Lunch Program, although only for two data points (school years 1991–92 and 1998–99) (27). Energy content has remained fairly constant for lunches (with an increase of 3% in primary schools and a decrease of 3% in secondary schools) but declined somewhat for breakfast (a decrease of 4% for primary schools and a decrease of 10% for secondary schools). Fat content declined and was replaced by carbohydrates with no change in protein content (27). The overall role of school diet in children's diet is less clear because the data refer to meals offered, not necessarily consumed, and because there have been large increases in participation in the School Breakfast Program. Schools provided approximately 8% of all meals and snacks and contributed 9% of total calories for children aged two to 19 years in 1994–1996, but the importance of school foods in a child's diet was highest among children aged six to 11 years (28).

## Discussion

In 1995, "Obesity in Britain: Gluttony or Sloth?," a study published in the *BMJ*, energized the debate on whether the obesity epidemic is caused by declining physical activity or increasing energy intake (29). The authors came down on the "sloth" side for adults. Even if one could separate energy intake and expenditure, we saw in this review that existing data are too limited to support a conclusive analysis. However, food consumption patterns have changed dramatically while youth physical activity patterns have not. In fact, the data generally show fewer changes in physical activity — changes in time studying at home, participating in exercise, or taking part in high school PE — than commonly thought. Changes went in the opposite direction, even if at a minimal level — less television watching and more active transportation time. These patterns do not exclude the possibility that overall physical activity has declined, because major changes in time use have occurred, and, for example, we have not determined activity levels in after-school and day-care settings where time spent by children has increased substantially. Both dietary and physical activity interventions can affect weight gain. Interventions affecting physical activity can be desirable even if recent increases in obesity among youth have been primarily related to changes in diet.

## Figures and Tables

**Table:** Percentage of Trips by Transportation Mode Among U.S. Children Aged Five to 15 Years^a^

	** 1977**	** 1990**	** 2001**
**Personal vehicles**	76.0	65.5	71.3
**Public transportation**	2.7	2.1	1.0
**School bus**	7.6	15.4	10.2
**Bicycle**	1.3	2.3	3.3
**Walk**	11.9	14.1	13.3
**Other**	0.4	0.4	0.9

a Data based on National Personal Transportation Surveys for 1977 and 1990 and the National Household Travel Survey for 2001 (9).

## References

[1] Ogden CL, Flegal KM, Carroll MD, Johnson CL (2002). Prevalence and trends in overweight among US children and adolescents,1999-2000. JAMA.

[2] Hill JO, Peters JC (1998). Environmental contributions to the obesity epidemic. Science.

[3] Hill JO, Wyatt HR, Reed GW, Peters JC (2003). Obesity and the environment: where do we go from here?. Science.

[4] Ewing R, Schmid T, Killingsworth R, Zlot A, Raudenbush S (2003). Relationship between urban sprawl and physical activity, obesity, and morbidity. Am J Health Promot.

[5] Sturm R, Cohen D (2004). Suburban sprawl and physical and mental health. Public Health.

[6] Koplan JP, Liverman CT, Kraak VA Preventing childhood obesity: health in the balance.

[7] U.S. Department of Agriculture (2004). Food consumption data system [Internet].

[8] Robinson JP, Godbey GG (1999). Time for life: the surprising ways Americans use their time.

[9] Bureau of Transportation Statistics National household travel survey [Internet].

[10] Porter RC (1999). Economics at the wheel: the costs of cars and drivers.

[11] Carter RC (2002). The impact of public schools on childhood obesity. JAMA.

[12] Centers for Disease Control and Prevention (1997). Guidelines for school and community programs to promote lifelong physical activity among young people. MMWR Recomm Rep.

[13] Datar A, Sturm R (2004). Physical education in elementary school and body mass index: evidence from the early childhood longitudinal study. Am J Public Health.

[14] Centers for Disease Control and Prevention (2002). Youth Risk Behavior Surveillance System (YRBSS): United States, 2001. MMWR Morb Mortal Wkly Rep.

[15] Enns CW, Mickle SJ, Goldman JD (2002). Trends in food and nutrient intakes by children in the United States. Family Economics and Nutrition Review.

[16] Nielsen SJ, Siega-Riz AM, Popkin BM (2002). Trends in energy intake in U.S. between 1977 and 1996: similar shifts seen across age groups. Obes Res.

[17] Jahns L, Siega-Riz AM, Popkin BM (2001). The increasing prevalence of snacking among US children from 1977 to 1996. J Pediatr.

[18] Nielsen SJ, Popkin BM (2003). Patterns and trends in food portion sizes, 1977-1998. JAMA.

[19] Bray GA, Nielsen SJ, Popkin BM (2004). Consumption of high-fructose corn syrup in beverages may play a role in the epidemic of obesity. Am J Clin Nutr.

[20] Popkin BM, Nielsen SJ (2003). The sweetening of the world's diet. Obes Res.

[21] Ludwig DS, Peterson KE, Gortmaker SL (2001). Relation between consumption of sugar-sweetened drinks and childhood obesity: A prospective observational analysis. Lancet.

[22] Harnack L, Stang J, Story M (1999). Soft drink consumption among U.S. children and adolescents: nutritional consequences. J Am Diet Assoc.

[23] Cullen KW, Ash DM, Warneke C, de Moor C (2002). Intake of soft drinks, fruit-flavored beverages, and fruit and vegetables by children in grades 4 through 6. Am J Public Health.

[24] Pereira MA, Jacobs DR, Van Horn L, Slattery ML, Kartashov AI, Ludwig DS (2002). Dairy consumption, obesity, and the insulin resistance syndrome in young adults: the CARDIA Study. JAMA.

[25] Lin BH, Guthrie J, Frazão E (undated). Nutrient contribution of food away from home. America's eating habits: changes and consequences.

[26] Akerlof GA (1970). The market for 'lemons': quality uncertainty and the market mechanism. Quarterly Journal of Economics.

[27] U.S. Department of Agriculture, Food and Nutrition Service (2001). School Nutrition Dietary Assessment Study II. Special Nutrition Programs Report No. CN-01-SNDAIIFR.

[28] Lin BH, Guthrie J, Frazão E (1999). Quality of children's diets at and away from home: 1994-96. Food Review.

[29] Prentice AM, Jebb SA (1995). Obesity in Britain: gluttony or sloth?. BMJ.

